# Parasites in food webs: the ultimate missing links

**DOI:** 10.1111/j.1461-0248.2008.01174.x

**Published:** 2008-06

**Authors:** Kevin D Lafferty, Stefano Allesina, Matias Arim, Cherie J Briggs, Giulio De Leo, Andrew P Dobson, Jennifer A Dunne, Pieter T J Johnson, Armand M Kuris, David J Marcogliese, Neo D Martinez, Jane Memmott, Pablo A Marquet, John P McLaughlin, Erin A Mordecai, Mercedes Pascual, Robert Poulin, David W Thieltges

**Affiliations:** 1 Western Ecological Research Center, U.S. Geological Survey. c/o Marine Science Institute UC, Santa Barbara, CA 93106, USA; 2 National Center for Ecological Analysis and Synthesis UC, Santa Barbara, CA 93106, USA; 3 Sección Zoología Vertebrados, Facultad de Ciencias, Univ. República Uruguay, Iguá 4225 Piso 9 Sur, Montevideo, Uruguay; 4 Center for Advanced Studies in Ecology and Biodiversity (CASEB) and Departamento de Ecología, Pontificia Univ. Católica de Chile Casilla 114-D, Santiago, Chile; 5 Ecology, Evolution and Marine Biology UC, Santa Barbara, CA 93106, USA; 6 Dipartimento di Scienze Ambientali, Univ. degli Studi di Parma 43100 Parma, Italy; 7 Department of Ecology and Evolutionary Biology, Eno Hall, Princeton University Princeton, NJ 08544-1003, USA; 8 Santa Fe Institute Santa Fe, NM 87501, USA; 9 Pacific Ecoinformatics and Computational Ecology Lab Berkeley, CA 94703, USA; 10 Ecology and Evolutionary Biology, University of Colorado Boulder, CO 80309, USA; 11 Environment Canada, St Lawrence Centre 105 McGill, 7th Floor, Montreal, Quebec, Canada H2Y 2E7; 12 School of Biological Sciences, University of Bristol Bristol BS8 3PZ, UK; 13 University of Michigan 2045 Kraus Natural Science Bldg. 830 N. University, Ann Arbor, MI 48109-1048, USA; 14 Instituto de Ecologia y Biodiversidad (IEB) Casilla 653, Santiago, Chile; 15 Department of Zoology, University of Otago PO Box 56, Dunedin 9054, New Zealand

**Keywords:** Disease, food web network, parasite

## Abstract

Parasitism is the most common consumer strategy among organisms, yet only recently has there been a call for the inclusion of infectious disease agents in food webs. The value of this effort hinges on whether parasites affect food-web properties. Increasing evidence suggests that parasites have the potential to uniquely alter food-web topology in terms of chain length, connectance and robustness. In addition, parasites might affect food-web stability, interaction strength and energy flow. Food-web structure also affects infectious disease dynamics because parasites depend on the ecological networks in which they live. Empirically, incorporating parasites into food webs is straightforward. We may start with existing food webs and add parasites as nodes, or we may try to build food webs around systems for which we already have a good understanding of infectious processes. In the future, perhaps researchers will add parasites while they construct food webs. Less clear is how food-web theory can accommodate parasites. This is a deep and central problem in theoretical biology and applied mathematics. For instance, is representing parasites with complex life cycles as a single node equivalent to representing other species with ontogenetic niche shifts as a single node? Can parasitism fit into fundamental frameworks such as the niche model? Can we integrate infectious disease models into the emerging field of dynamic food-web modelling? Future progress will benefit from interdisciplinary collaborations between ecologists and infectious disease biologists.

## Introduction

Think ‘food web’ and the African Savannah may come to mind. Even children recognize that zebras eat grass and lions eat zebras. Less obvious, however, are the 54 or more consumers that eat lions, which include lions themselves, leopards, hyenas and a notable diversity of infectious agents (or parasites): two arthropods, two bacteria, 31 helminths, six protozoans and 10 viruses (Nunn & Altizer 2005).

The strong impacts of some infectious agents in food webs have been apparent for over a hundred years. After 1889, the introduced rinderpest virus rapidly reduced the ungulates of the African Savannahs to 20% of their original abundance (Sinclair 1979). Without prey, carnivores starved and their populations declined. Freed from grazing, the grass grew tall, which increased the frequency of fire and, in turn, reduced resources for tree-feeding species such as giraffes (Sinclair 1979). Similar stories exist for other systems. The accidental invasion of myxomatosis into Great Britain in 1953 led to shifts in vegetation, predators and ants, as well as the indirect extinction of a butterfly (Sumption & Flowerdew 1985). In the 1980s, epidemic mortality (98% loss) of the Caribbean black-spined sea urchin (*Diadema antillarum*) (Lessios 1988), a keystone herbivore, shifted the reef system from coral-dominated to algae-dominated (Hughes 1994). Similarly, recent mass mortalities of black abalone (Lafferty & Kuris 1993) from a rickettsia (intracellular bacterium) have permitted the colonization of fouling organisms, altering the iconic rocky intertidal communities of southern California (Miner *et al.* 2006).

Given that food webs are central to fundamental ecological concepts such as the stability, diversity and complexity of ecosystems (Pascual & Dunne 2006), it is important to understand the influence that parasites may have on the structure, dynamics and function of food webs. As discussed below, parasites can augment the flow of energy, alter the strength of interactions, change productivity and cause trophic cascades. The inclusion of infectious agents in this fundamental ecological concept might allow for a better understanding, evaluation and mitigation of human impacts on ecosystems, including biodiversity loss, climate change, exotic species, pollution, bioremediation, pest control and fishery exploitation. For instance, in California, an invasive Japanese mud snail, *Batillaria attramentaria*, replaced a native snail so similar that that food-web dynamics appear unchanged after the invasion; yet, the invasion led to the loss of more than a dozen native trematode parasites and the addition of a Japanese trematode, with potentially important consequences for the birds, fishes and invertebrates that also serve as hosts for trematodes (Torchin *et al.* 2005).

There is nothing conceptual about food webs that precludes the inclusion of parasites; however, most food-web datasets either lack or under-represent parasites, despite numerous demonstrations of their importance, as well as calls for greater inclusion and higher resolution of all types of taxa in food webs (Marcogliese & Cone 1997; Borer 2002). The main reason parasites are missing from food webs is that researchers tend to compile data on the easy-to-observe species in ecosystems. Small, cryptic or non-free-living organisms, such as prokaryotes, soil organisms and parasites, are generally absent from food webs. This is partly attributable to a lack of disciplinary integration. The parasitology skills necessary to recognize and quantify parasites (often having complex life cycles with morphologically distinct stages) differ from the skills of the ecologists who usually compile food webs from predator–prey and herbivore–primary producer links. We note that two parasitic functional groups, insect parasitoids and herbivores that feed non-lethally on plants, are common in some food webs, probably because they are relatively easy to quantify. Leaving parasites out of food webs restricts our understanding to the ‘free-living’ portion of ecosystems, and thus reduces to a fraction the number of species in the networks (i.e. < 50% of the metazoans; Price 1980). It also excludes the potential effects of parasites on their hosts. Not surprisingly, the theoretical and empirical approaches of community ecology related to predator–prey dynamics and food-web research have developed separately and differently from the theory and approaches used in parasitology and host–parasite dynamics.

Food-web ecology, and related theory, has gone through a significant transition, progressing from the analysis of highly aggregated and unevenly resolved data to more evenly and highly resolved data (see Pascual & Dunne 2006 for a review of the terminology and key topics related to food webs). For example, recently compiled data tend to more accurately represent the levels of cannibalism, omnivory and intraguild predation seen in natural systems than were available in earlier datasets (e.g. Martinez 1991; Polis 1991). We suggest that researchers are on the verge of another step of significant improvement – the systematic inclusion of parasites in food-web data and analysis (Huxham *et al.* 1995; Thompson *et al.* 2005; Lafferty *et al.* 2006a). In this review, we consider the range of parasite life cycles and their roles as consumers and resources, and we summarize existing information on the role of parasites in food-web topology and dynamics, identifying current challenges. Looking to the future, we consider possible ways to include parasites in food webs and food-web theory. Finally, we discuss how food webs may provide important insights into infectious disease dynamics.

## How do parasites fit into food webs?

### Parasites as consumers

Before putting parasites into food webs, it is prudent to consider how parasitism differs from predation as a trophic strategy, and how parasites differ from each other. Parasites may affect hosts differently than predators affect prey. While a predator kills multiple prey individuals during its life, a parasite obtains nourishment from a single host during a life stage. A further dichotomy separates pathogens (microparasites) from typical parasites (macroparasites). Pathogens (modelled as microparasites) multiply within or on a host, and the outcome of infection generally depends on the success of the host response (Anderson & May 1979). The impact of a pathogen is typically intensity-*independent* because a single infection event, and the parasite's subsequent within-host production, yields the full array of pathology for the host. In contrast, typical parasites (modelled as macroparasites) are intensity dependent. Their impact on the host increases with the number of parasites in the host, each of which represents an independent infection event. The distributions of macroparasites within a host population tend to be highly aggregated such that only a few hosts bear most of the disease burden.

With the advent of cooking meat, humans largely escaped their exposure to parasites through food. A few food-borne parasites acquired through sashimi and steak tartar are a pale reminder of what our ancestors contended with on the African Savannahs. In most of the animal world, however, trophically transmitted parasites remain particularly important to food-web structure. Their life cycles follow predator–prey linkages as final hosts consume infected intermediate hosts. Many trophically transmitted hosts are strong behaviour modifiers, thereby increasing predation on infected prey hosts (Moore 2002). Hence, they may increase interaction strength, sometimes substantially (Lafferty & Morris 1996). Similarly, parasites may facilitate new trophic interactions. For example, one trematode species causes its cockle host to strand itself on the sediment surface, where fish consume the exposed cockle's foot and become infected with the parasite (Mouritsen & Poulin 2003). Other opportunistic predators, such as whelks, exploit surface-stranded cockles, adding to the total cockle biomass diverted towards other members of the food web because of parasitism.

Parasitoids have a unique consumer strategy. Although the consequence of an infection with typical parasites and pathogens is usually non-lethal, parasitoids (including many parasitic wasps) necessarily kill one and only one host with extremely efficient energy conversion. As a result, their body size is also large compared with that of their hosts (Kuris 1974). Parasitic castrators likewise reduce host fitness to zero; however, unlike the hosts of parasitoids, the castrated hosts live on to consume resources and potentially compete with their uninfected counterparts (Lafferty 1993). Many parasitic castrators do not affect host longevity and may even enhance it by reducing mortality risks for infected hosts. Some alter host behaviour, morphology and/or growth in such profound ways that they create a distinctive niche for the castrated organisms, governed, in large part, by the genotype of the castrator (Miura *et al.* 2006).

Finally, it is important to recognize and evaluate micropredators. Although they are not infectious, micropredators such as mosquitoes, leeches, browsers and grazers, attack more than one host (similar to a predator), but impact that host in an intensity-dependent manner, similar to a typical parasite. All of these and a few other distinctive types of consumers have categorical definitions (Lafferty & Kuris 2002). Therefore, although we broadly consider parasites in food webs here, we do so with the knowledge that there are many parasitic strategies and that the differences among these strategies may influence food-web dynamics.

### What is the trophic level of a parasite?

**Figure 1:**
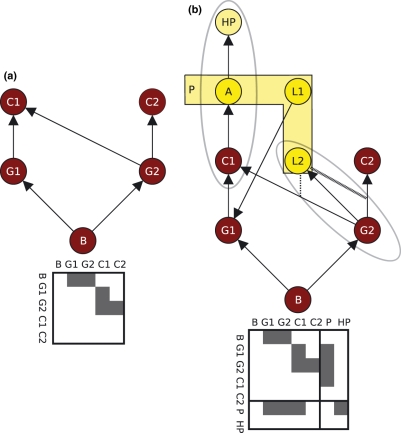
Graphical depiction of a simple five-node food web before (a) and after (b) adding two parasites. Taxa represented are basal (B), grazer (G1, G2), predator (C1, C2), parasite (P) and hyperparasite (HP). The parasite (P) has an adult stage (A) using C1 as a host, a free-living larval stage (L1) and a parasitic larval stage (L2) in an intermediate host (G2). Transmission from intermediate host to final host occurs when a final host eats an infected intermediate host. The yellow L-shaped box contains the three life stages (yellow circles) of the parasite, P. Ellipsoids indicate parasites occurring within hosts. Arrows represent feeding links with the arrow pointing from the resource to the consumer (depicting energy flow). There are three types of predator–parasite links (dashed lines): feeding on the free-living stage of a parasite (L1–G1), ingestion of an infected intermediate host with the possibility of transmission of the parasite to the predator (L2–C1) and incidental ingestion of a parasite in an infected prey (L2–C2); the latter two we merge with predator prey links. Below the stick and ball figures are who eats whom matrices where consumers are rows and resources are columns. The matrix in (b) has four quadrants, clockwise from the top left, predator–prey, predator–parasite, parasite–parasite and parasite–host. Note that in the matrix of the free-living web, 20% of the possible links (directed connectance) are present while after adding parasites this increases to 24.5%– but only if predator–parasite and parasite–parasite links are included. This is also substantially higher than the 14% (7/49) predicted if the number of conventional links were to scale with the square root of possible interactions. Also, note that while the parasite P feeds on two hosts, it is not a generalist because it requires both to persist.

In the simplest sense, a consumer is one trophic level above its resource. Many species feed at more than one trophic level, and food-web topology can provide various measures of the trophic level of omnivores (Williams & Martinez 2004). Like omnivores, parasites with complex life cycles may feed on several different trophic levels (Fig. 1) but, unlike conventional omnivores, their omnivory occurs across distinct life stages.

The ratio of heavy nitrogen-15 to light nitrogen-14 (δ^15^N) can indicate an organism's trophic position, but this may not work well for parasites. Although predators are almost always ^15^N-enriched compared with their prey, parasites (Table 1) are sometimes ^15^N-depleted compared with their hosts (Pinnegar *et al.* 2001). Other parasites have a similar enrichment to their hosts, whereas a few parasites are more enriched than expected for a direct consumer (O’Grady & Dearing 2006). The level of enrichment can even vary between parasite taxa within hosts. Intestinal nematodes parasitizing rabbits are ^15^N-enriched whereas intestinal cestodes in the same host species are ^15^N-depleted (Boag *et al.* 1998; Neilson *et al.* 2005). Further complicating matters, different parasite species on the same host or the same parasite species on different hosts can differ in their isotope enrichment (Deudero *et al.* 2002). This difference in ^15^N between predators and parasites likely stems from the fact that parasites are relatively selective in which parts of the host they consume. For instance, some parasites may feed on intestinal contents rather than on host tissue; others selectively absorb particular biochemical compounds such as amino acids, live in and feed on different host tissues, or have altered metabolism that varies with life stage (Pinnegar *et al.* 2001; Deudero *et al.* 2002). For these reasons, a topological assessment seems the best approach for determining trophic level, as long as the specification considers variation among life stages.

**Table 1:** Summary of stable isotopes ratios (δ^15^N) for parasite-host relationships

Parasite taxon	Host	^15^N	Reference
Trematoda	Fish	Similar	Iken *et al.* (2001)
Cestoda	Mammalia	Depleted	Boag *et al.* (1998), Neilson *et al.* (2005)
	Fish	Depleted	Deudero *et al.* (2002), Persson *et al.* (2007)
Nematoda	Mammals	Enriched	Boag *et al.* (1998), Neilson *et al.* (2005)
	Fish	Enriched	Pinnegar *et al.* (2001)
	Fish	Depleted	Iken *et al.* (2001), Deudero *et al.* (2002)
	Reptiles	Similar	O’Grady & Dearing (2006)
	Reptiles	Enriched	O’Grady & Dearing (2006)
Copepoda	Fish	Depleted	Pinnegar *et al.* (2001), Deudero *et al.* (2002)
	Fish	Enriched	Iken *et al.* (2001), Deudero *et al.* (2002)
Isopoda	Fish	Depleted	Iken *et al.* (2001)
		Similar	Pinnegar *et al.* (2001)
Cirripeda	Decapods	Similar	Iken *et al.* (2001)
Insecta	Insects	Enriched	Doucett *et al.* (1999)
	Mammals	Enriched	Boag *et al.* (1998), Voigt & Kelm (2006)
Gastropoda	Holothurians	Similar	Iken *et al.* (2001)

Parasites can be ^15^N-enriched (the parasite is at a higher trophic level than its host), similar in trophic level or ^15^N-depleted (the parasite is at a lower trophic level than its host).

## How do we add parasites to food webs?

It is possible to compile integrated food webs *de novo* (Lafferty *et al.* 2006b). Thus, when constructing a new food web, parasites could be incorporated as a matter of course. This, we hope, will be the future standard. Until then, it seems possible to add parasite information to many existing community food webs. The primary literature contains a wealth of information on parasite–host records, and new online databases provide convenient summary information on parasites searchable by host species (e.g. the London Natural History Museum's database of 470 000 host–parasite records at http://www.nhm.ac.uk/research-curation/projects/host-parasites/database/). Not all available food-web datasets are appropriate for expansion. Some have taxonomic categories that are highly aggregated by functional roles rather than by taxonomic position (e.g. Polis 1991), while others are dominated by taxa with scant information about parasites. Webs used in various recent comparative analyses and models of food-web structure (Williams & Martinez 2000; Dunne *et al.* 2002b, 2004) would be particularly suitable for the integration of parasites. Sometimes authors consider networks of hosts and parasites in a narrower context than a community food web (Martinez *et al.* 1999; Muller *et al.* 1999; Memmott *et al.* 2000; Rott & Godfray 2000; Vazquez *et al.* 2007; Mouillot *et al.* 2008). One could expand these narrowly focussed webs to include a full set of the free-living taxa in the community and a full set of parasites.

Although we advocate improving the detail of food webs by adding parasites, it is clearly intractable to include every species in a system. All food webs set boundaries for what species to include or exclude. For parasites, as for free-living species, this will often come down to the quality of the data. Systematic and equitable consideration of parasites for all free-living species in the food web would be ideal, but information on parasites will invariably be more detailed for some host groups than for others, opening the potential for bias due to uneven inclusion or resolution of taxa. Researchers should decide to either include or exclude parasites that have, as part of their life cycle, stages outside the defined spatial or temporal scope of the food web (it is easier to include at first and exclude later, if necessary). In particular, if the known list of parasites from a host species includes parasites described from distant locations that might not occur within a particular study area, food webs based on such lists could overestimate parasite diversity in a particular web.

## Can parasites inform free-living links?

**Figure 2:**
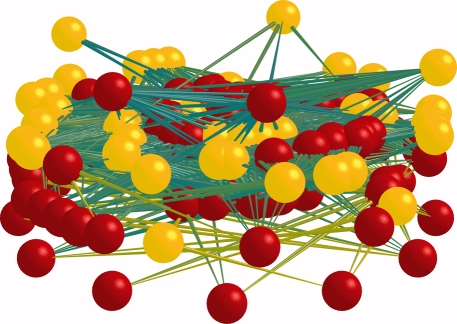
Three-dimensional visualization of the complexity of real food webs with parasites using data from the Carpinteria Salt Marsh Web (Lafferty *et al.* 2006b). Image produced with software available from the Pacific Ecoinformatics and Computational Ecology Lab, http://www.foodwebs.org. Balls are nodes that represent species. Parasites are the light-shaded balls and free-living species are the dark-shaded balls. Sticks are the links that connect balls through consumption. Basal trophic levels are on the bottom; upper trophic levels are on the top.

The process of adding parasites to food webs can inform predator–prey interactions, thereby improving the free-living links in the web. Such information is valuable because knowledge of who eats whom is often anecdotal, based on sparse observations, or determined by gut contents (which underestimate soft-bodied prey). Trophically transmitted parasites provide natural biological indicators of trophic links between organisms within ecosystems (reviewed in Marcogliese & Cone 1997; Marcogliese 2003). In comparison to gut contents, which offer insights into a very limited temporal window of feeding activity, trophically transmitted parasite assemblages are the accumulated consequence of long-term feeding by their hosts. Sometimes, parasites may reveal the existence of diet items not ascertainable from gut contents such as fragile and quickly digested food items (e.g. soft-bodied zooplankton). For example, the analysis of parasites reveals the diets of brook charr more precisely than does examination of stomach contents (Bertrand *et al.* 2008). In addition to indicating what a host ate, larval parasites in a host can reflect the type of predators that might eat the host (Huxham *et al.* 1995; Marcogliese 2003). Knowledge of parasites allowed Lafferty *et al.* (2006b) to add several predator–prey links to an estuarine food web (Fig. 2). For example, the trematode *Cloacitrema michiganensis* parasitizes American Coots. These waterfowl were thought to forage exclusively on vegetation. However, because the trematode encysts on opercula of the horn snail, *Cerithidea californica*, American Coots evidently include *C. californica* in their diet (they probably ingest small snails when feeding on vegetation).

## Do parasites affect food-web topology?

The paucity of food webs with parasites makes it difficult to fully answer this question. Initial efforts to add parasites to food webs revealed the intuitive effects of increases in species richness, link number, trophic level and chain length (Fig. 1; Huxham *et al.* 1995; Thompson *et al.* 2005). However, these webs were not fully resolved. Furthermore, the methods used underestimated the effect of parasites on one key food-web metric, connectance, because they excluded the existence of parasite–parasite or predator–parasite links from the numerator of connectance (but not from the denominator). Correcting this calculation suggests that parasites increase connectance (Lafferty *et al.* 2006a). Of the webs with parasites, the Carpinteria Salt Marsh web is the most finely resolved. Including parasites in this web doubles connectance and quadruples the number of links, more than 75% of which include parasites (Lafferty *et al.* 2006a). While the vulnerability of species to predators decreases as trophic level increases, inserting parasites into the Carpinteria Salt Marsh food web (Fig. 2) disproportionately raises the number of natural enemies for higher trophic-level species. This suggests that intermediate trophic levels are most vulnerable to the full range of natural enemies (Lafferty *et al.* 2006a). Evaluating the generality of these findings will require examination of datasets from other habitats with an equivalent resolution of both free-living and parasite taxa. Other analyses can help determine the effects of parasites on food-web structure. For instance, one could ask whether the normalized cumulative link distributions for parasites and predators fall along similar universal curves (Dunne *et al.* 2002a), whether the webs with and without parasites display similar motifs (patterns of connections within the network) (Stouffer *et al.* 2007), or how these motifs relate to parasite transmission and persistence.

Many parasites are highly specialized and should be sensitive to the loss of host species. Adding such species to food webs can reduce the robustness of a food web because they are highly susceptible to secondary extinction if their host resources go extinct (Fig. 1; Dunne *et al.* 2002b). While parasite loss is unlikely to elicit as much sympathy as the loss of more charismatic species, the rapid disappearance of parasites from networks in the face of perturbation may make parasites especially useful as indicator species. Their ecological and taxonomic diversity, and their ubiquity in terrestrial and aquatic systems, suggest that parasites may provide a smorgasbord of potential bioindicators for use by environmental scientists. The loss of particular parasites or unique combinations of parasites can clearly indicate specific habitat ills. For example, trematode communities in snails provide an effective and reliable metric of ecological restoration in an estuary (Huspeni & Lafferty 2004). Including parasites more systematically in food-web data is likely to increase their utility as indicators.

## Do parasites affect food-web dynamics?

Although parasites may affect network topology, there is scant information on how parasites affect many aspects of ecological dynamics, including variation in abundance among species and flows along links. Investigations into the dynamics of complex ecological networks use a variety of approaches, most recently involving nonlinear bioenergetic models (Brose *et al.* 2006b). Compared with other consumers, parasites may have different metabolic scaling coefficients, functional responses and connections to other species. These variables are critical determinants of the behaviour of bioenergetic food-web models. In addition, the diversity of species that consume a parasite, a topological measure of the ‘vulnerability’ of a species, may differ from that of similarly sized non-parasitic species because parasites are protected from many predators while living inside their hosts.

There are few empirical data on the magnitude of parasitic influences on energy flow within real food webs. However, recent work indicates that parasites comprise a substantial fraction of the biomass and production in estuarine ecosystems (Kuris *et al.* 2008). Energy flows directly from hosts to parasites. Based on metabolic scaling coefficients and field data on infection levels, it might be possible to compute both the mean rate of energy flow and its variance (determined by the level of aggregation of the parasites among their hosts) for key host–parasite links in a given food web. Parasites can also modulate the flow of energy along other trophic links. For example, as mentioned previously, trophically transmitted parasites modify the behaviour of hosts in ways that increase their rates of consumption by predators. Field experiments used to quantify these rates for both parasitized and unparasitized prey have shown that parasites can increase rates of energy flow along certain trophic links (Lafferty & Morris 1996; Thomas & Poulin 1998). For example, killifish are common forage species for birds in California estuaries; in locations where they are infected by a common brain-encysting trematode, they are 10–30 times more likely to be fed on by the birds that serve as final hosts to the worm (Lafferty & Morris 1996). Obtaining this sort of quantitative data on the influence of parasitism on energy flow through food webs will be a challenging but necessary step in moving beyond the simple effects of parasites on topology.

The effect of diversity on stability is probably the most studied aspect of food-web dynamics. Simple early models predicted that increased species diversity and complexity decrease network stability (May 1973). This suggests that the increases in species diversity and connectance achieved by adding parasites will destabilize network dynamics. Although more recent models find that diversity can increase stability when consumers are larger than their resource species (Brose *et al.* 2006a), parasites are smaller than their hosts, and inclusion of parasites could result in network instability if unstable parasite–host feeding links overwhelm more stable predatory–prey dynamics (Otto *et al.* 2007). In addition, adding parasites to food webs extends the length of trophic chains (Williams & Martinez 2004), which decreases food-web stability. However, the addition of long loops of weak interactions that may be the characteristic of parasites with complex life cycles might offset the destabilizing effects of increased connectance (Neutel *et al.* 2002). Further, pathogens shared between host species may be strongly stabilizing, particularly as their dynamics are inherently frequency dependent, with the commonest host species suffering disproportionately from pathogens (Dobson 2004).

Diversion of energy from hosts to parasites could affect food-web stability because the stability of predatory, competitive and intraguild predation interactions depend on the efficiency of resource exploitation (Borer *et al.* 2007). Parasites reduce the extent to which consumers can apply acquired energy to their own needs, thereby reducing the efficiency of energy transfer from prey to predator (Wood *et al.* 2007). Infected hosts often increase their metabolic rate compared with their unparasitized counterparts (Morand & Harvey 2000) because, at a minimum, infected hosts have repair costs and increased defensive costs. In addition, infected hosts may be sufficiently impaired that their feeding rate and efficiency suffers (Wood *et al.* 2007). The efficiency of energy transfer ultimately constrains food-chain length, limiting top predators (Arim *et al.* 2007). Parasites, therefore, could limit the abundance and diversity of top predators, essentially eroding the trophic pyramid from within. However, by slowing the growth rate of top predators, parasites could prevent extinction-inducing oscillations in predator abundance (Otto *et al.* 2007). Clearly, the potential effect of parasites on stability is a complex and unresolved issue.

## How can we expand food-web theory to accommodate parasites?

Present approaches to constructing food webs may not adequately capture important elements of the complex life cycles of many parasites (Fig. 1). This is not a challenge unique to parasites because many free-living species also have complex life cycles. Lumping parasite life stages into a single node might inflate connectance and robustness, making a species that specializes at one or more stages appear to be a generalist feeder (and, therefore, robust to secondary extinction). Dividing a species into several nodes illustrates how specialization within a stage subjects a parasite to a higher chance of secondary extinction. For this reason, before calculating topological statistics of networks, the implications of life stages require careful consideration. Ideally, a network would identify the unique trophic connections for each distinct life stage, while also maintaining the identity of species through growth or ontogenetic links, perhaps by considering growth and feeding as orthogonal modes of energy flow through a network. Expanding networks beyond two dimensions will, however, be challenging as the typical tools to deal with networks (linear algebra, graph theory, circuits theory) usually consider only one type of interaction.

It is not clear how food webs should represent the fact that parasites are also prey (Fig. 1, Box 1). Some analyses of the effects of parasites on food-web topology acknowledge that free-living stages of parasites may be subject to predation (e.g. Thompson *et al.* 2005). For instance, new studies indicate that a wide diversity of vertebrate and invertebrate predators eat free-living stages of parasites (Lafferty *et al.* 2006a; Schotthoefer *et al.* 2007; Thieltges *et al.* 2008). Some predators specialize on larval and adult parasites. For example, cleaner shrimps and fishes pick ectoparasites from fish hosts (Grutter 1999) and oxpeckers perching on African mammals prey on ticks, botfly larvae and other ectoparasites (Fry *et al.* 2000). We do not know the consequences of this type of predation on parasite transmission or food-web dynamics but, in some systems, the biomass and productivity of these stages may be substantial (Kagami *et al.* 2007). Less obvious is that predators consume the parasites in their prey items (Fig. 1). Hence, most of the links involving parasites may also be predator–parasite links that strongly affect food-web topology; however, it is unclear if or how to accommodate such links (Lafferty *et al.* 2006a). Such predator–parasite links are akin to incidental predation (the rabbit that inadvertently eats an unlucky ant on a blade of grass) in that they may matter little for the transfer of energy to consumers. However, predation on infected prey could amount to substantial losses to parasites. Predation also represents a key transmission pathway, and predators often acquire trophically transmitted parasites from their prey, including a variety of helminths and some microparasites. For instance, in the Carpinteria Salt Marsh web, a third of the parasite species in prey consumed by predators can use the predator as a host (Lafferty *et al.* 2006b).

**Box 1 How to include parasite data into food webs**

Topological food webs consist of an *N*-by-*N* matrix of *n* species, in which the predators occur in rows and the prey occur in columns (Fig. 1). Binary entries in the cells of the matrix (e.g. 0 or 1) indicate the presence or absence of predator–prey links. The simplest way of adding parasites to such topological webs is to add additional rows with the parasite species present in the food web. In these additional rows, binary entries indicate parasite–host links. Hence, the columns now represent hosts and prey (Huxham *et al.* 1995; Thompson *et al.* 2005). However, there are additional types of food-web links involving parasites, predator–parasite and parasite–parasite (Fig. 1; Lafferty *et al.* 2006a). Predator–parasite links arise from predation on free-living stages of parasites and on the hosts of parasites. The latter may either lead to successful transmission or parasite death if the predator is an unsuitable host. Parasite–parasite interactions mainly result from intraguild predation among parasite species within their hosts or from hyperparasitism. Adding these different types of links creates four sub-webs: predator–prey, parasite–host, predator–parasite and parasite–parasite (Fig. 1). One can analyse the different sub-webs separately or combine them for more complex analyses. However, how this can be carried out remains an open question. Weighting links by interaction strength is a challenging proposition for any food web (e.g. Cohen *et al.* 1990a), and unexplored for food webs with parasites.

To understand whether parasites affect food-web structure, one must consider the definitions and determinations of structure in general. To better understand how real food webs differ from random assemblages of nodes and links, several models combine stochastic elements with simple link assignment rules to generate and predict the network structure of empirical food webs (Cohen & Newman 1985; Williams & Martinez 2000). These models share a basic formulation based on predator–prey interactions (Williams & Martinez 2000). There are two empirically quantifiable parameters: (i) *S*, the number of trophic species in a food web, and (ii) *C*, the connectance of a food web, defined as observed links divided by possible links, or *L* /*S*^2^ (Fig. 1). Each species is assigned a ‘niche value’*n*_*i*_ drawn randomly and uniformly from the interval [0,1]. As *n*_*i*_ increases, the generality (i.e. the number of prey) of species also increases. The models differ in the rules used to distribute links among species. For example, in the cascade model (Cohen & Newman 1985) as modified by Williams & Martinez (2000), each species has the fixed probability *P* = 2*CS*/(*S*− 1) of consuming species with niche values less than its own, creating a food web with strict hierarchical feeding. The niche model (Williams & Martinez 2000) relaxes the hierarchy assumption and introduces a ‘feeding contiguity’ rule – each species consumes all species within a segment of the [0,1] interval. The arrangement of consumer and resource species along the interval may reflect their size, metabolic rate or trophic position (Stouffer *et al.* 2005). Having the centre of feeding ranges fall at or below the consumer's niche value ensures that species feed primarily on resources that are lower in the hierarchy (e.g. big species eat small species). In addition, having the size of feeding ranges grow in proportion to consumers’ positions in the hierarchy ensures that species with higher niche values are increasingly general in their feeding habits. The niche model, as well as two recent variants, the nested-hierarchy model (Cattin *et al.* 2004) and the generalized cascade model (Stouffer *et al.* 2005), do a much better job than the cascade model of generating structure similar to that seen in empirical food-web datasets (Stouffer *et al.* 2007). To date, models have not explicitly considered parasites, and inclusion of parasites violates assumptions of cascade models (Marcogliese & Cone 1997).

A central aspect of empirical food webs that tends to drive many aspects of structure is the balance of how general or specific taxa are in their feeding habits, and how vulnerable they are to consumption by one or more taxa (Schoener 1989). The extent to which parasites differ from predators in this regard may indicate how parasites will affect food webs. Link distribution histograms (frequency histograms of the number of links each species has) readily summarize and analyse patterns of generality (how many resources a consumer eats), vulnerability (how many consumers eat a resource) and total links (total number of consumer and resource links for each taxon). Predator–prey data tend to display exponential link (or ‘degree’) distributions that, when normalized for average links per species in a particular web, follow a roughly universal functional form (Camacho *et al.* 2002b; Dunne *et al.* 2002a). The niche model also produces exponential link distributions (Camacho *et al.* 2002a), driven by its use of the beta distribution to assign the widths of feeding ranges, (Stouffer *et al.* 2005), which partially explains the good match between the niche model (and recent variants) and empirical data.

The niche model may fail to describe food webs with parasites if parasites have different generality and/or vulnerability than do free-living species. This is the case for a web focussed on species endophytic to several co-occurring grasses, including many parasitoids (Martinez *et al.* 1999) and a Scotch Broom-based web that includes parasitoids and pathogens (Memmott *et al.* 2000). Here, the addition of many parasitoids, most of which have specialized feeding habits, moves the link distributions away from the less-skewed exponential or uniform distributions typically seen in datasets without parasitoids (Dunne *et al.* 2002a). However, this may not be a fair assessment of the niche model because each of the parasitoid webs is just a source web based on one or a few plant taxa. A comparative analysis of food-web structure would best be carried out with cumulative community food-web data that represent the feeding interactions among a full range of co-occurring taxa within a particular habitat over several years. Such analyses are only currently possible for a very small number of webs.

There is a need for new models that incorporate parasites and explicitly consider different types of links. Parasites tend to be smaller than their hosts, and large parasites ultimately have fewer sufficiently large hosts available to them compared with small parasites. For these reasons, a potential way to extend the niche model to accommodate parasites is to reverse two of the three main rules. The ranges that comprise hosts have their centres above the position of the parasite, and the size of the range is inversely proportional to the position of the parasite (C. Warren, M. Pascual, KD Lafferty, in prep.). These types of questions and the comparison of models to data and to each other might be addressed more rigorously with likelihood-based approaches (S. Allesina, in prep.). Likelihood-based approaches could also help explore how to modify models to obtain better performance, and to identify which species traits provide the best ordering of species along a niche axis given empirical food-web data. For example, an analysis could determine which ordering of species along the niche axis (whether according to their biomass, metabolic rate, body size, type of consumer interaction, trophic level or some other trait) produces the highest likelihood. This would help to identify the ecological principles that drive the trophic structuring of communities.

In the niche model, a single trait – its position on the niche axis – determines whether a species is going to be a prey item for another species (i.e. whether it is included in the diet range of the predator). The overlap between the diets of the predators, therefore, can be associated with a single trait (e.g. prey size). When this happens, a food web is said to be interval (Cohen *et al.* 1990b). Generally speaking, none of the food webs measured in the field are perfectly interval: there is no way of accounting for predators’ overlap using just one trait (Stouffer *et al.* 2006). Nevertheless, food webs seem to be very close to perfect intervality (Stouffer *et al.* 2006), accounting partially for the success of the niche model. Specialist parasites should not decrease the degree of intervality in food webs (or connected measures such as ‘triangulation’, a proxy measure for intervality), because of their very specialized diets. However, if parasites with complex life cycles are represented as a single node, they may appear to be generalists (as they parasitize different species at different trophic levels). For example, parasites strongly decreased ‘triangulation’ (the food web is moved further away from intervality when parasites are added) in the Ythan Estuary food web (Huxham *et al.* 1996).

Dynamic food-web models might better approximate infectious disease processes by incorporating microparasite and macroparasite modelling approaches (see Box 2). In a network, however, analytical solutions quickly become intractable. One potential means for condensing parameter space in such models is to consider allometric scaling (Box 3). On average, large-bodied organisms live longer, metabolize more slowly and achieve lower densities than do small-bodied organisms (see Marquet *et al.* 2005). Thus, knowledge of one parameter, such as body size, may provide information about others (e.g. mortality rate). Advances in the field of allometric scaling relationships (see West & Brown 2005) provide tools for approximating energy flow through a network as a function of the absolute and relative body masses of the species in the web (e.g. Brose *et al.* 2006a). In addition, knowledge of relative body sizes can help generate hierarchical links in theoretical food webs (as suggested by the niche model) by considering that consumers are more likely to consume small-bodied resources (Raffaelli *et al.* 2000). Unfortunately, metabolic scaling relationships developed for free-living species might not apply to parasites (Box 3).

**Box 2 Simple macroparasite models and the calculation of *R*_0_**

The simple macroparasite model considers a parasite such as a nematode, for which the effect of the parasite on the host depends on the number of parasites infecting the host. We elaborate on this model to indicate the many ways that food webs can affect parasite dynamics. Such a parasite does not reproduce within the host, instead releasing free-living infectious stages into the environment. The level of host infection increases only through contact with infectious stages. In this model, we assume that the parasite's life cycle involves only a single host species. The model keeps track of the total density of adult parasites, *P*, infecting the host population and the density of free-living infective stages, *W*.

The equation for the host population is:

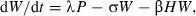

where *b* and *d* are the host birth and death rates, respectively (in a more realistic model, these parameters could be density dependent). The second term indicates that each parasite decreases the host birth rate by δ and increases the death rate by α, where *P*/*H* is the average density of parasites per host.

The equation for the free-living infectious stage is:

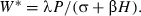

where λ is the rate at which parasites shed infective stages and σ the death rate of free-living infective stages. Infective stages encounter hosts at a transmission rate β, which results in new adult parasites. If the dynamics of *W* are fast compared with the lifespan of the host and parasites within the host, *W* simplifies to its equilibrium:

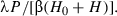

For convenience, we will set *H*_0_ = σ/β such that W^*^ becomes

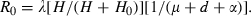

The equation for the density of parasites within hosts is:

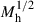

where μ is the death rate of adult parasites, and parasites within hosts suffer from any host mortality. The term in brackets accounts for the aggregated distribution of parasites across the host population, which occurs in most species modelled as macroparasites (where *k* is the clumping parameter of a negative binomial distribution).

*R*_0_ is the number of new parasites that an average parasite produces in an entirely susceptible host population. For the macroparasite model:

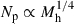

The first term in brackets is the fraction of the free-living infective stages released from a parasite that successfully infects a host, and the second term in brackets is the average lifespan of the parasite. Thus, increasing the host density, increasing the transmission rate, or decreasing any of the death rates of host or pathogen will lead to an increase in *R*_0_.

**Box 3 Allometric scaling for parasites in food webs**

Metabolic scaling may make it easier to add parasites to dynamic food-web models. The general metabolic scaling equation considers the effect of size and temperature upon the rate at which organisms process energy to sustain their biomasses as:
(1)
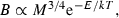

where *M* is the body mass, *B* the metabolic rate, *E* the activation energy, *k* the Boltzmann constant and *T* the temperature in Kelvin degrees (Brown *et al.* 2004).

A given amount of resources (*R*) in the environment will be able to sustain a maximum of *R*/*B* individuals per unit area. Hence, the maximum density of hosts (*N*_h_) should vary as a function of host mass (*M*_h_) as:
(2)
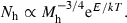

Equation 2 is equivalent to host carrying capacity, which increases *R*_0_ (see Box 2). Indeed, the threshold transmission rate of a parasite should scale with host mass as 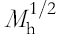 (DeLeo & Dobson 1996). The rate at which the parasite converts host resources to parasite biomass may be expressed as:
(3)
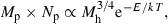

where *M*_p_ represents the mass of an individual parasite, and *N*_p_ is the number of parasites (George-Nascimento *et al.* 2004). Empirical data (George-Nascimento et al. 2004) suggest that for the case of helminth endoparasites, without considering the effect of temperature, 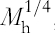, hence parasite density (the number of parasite per host gram) should vary as:
(4)
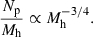

For the case of endoparasites, eqns 3 and 4 have been shown to hold. However, when all metazoan parasites (ecto and endoparasites) have been considered, the exponent in eqn 3 has been found to be close to unity (Poulin & George-Nascimento 2007).

## What can food webs tell us about parasites?

To this point, we have considered whether parasites are important elements of food webs and whether their inclusion might help us better understand food webs. An equally interesting perspective is that food webs may help us better understand infectious disease dynamics. Box 2 presents an example of a model used to study infectious disease dynamics. Such models assume that all static parameters can represent all other species in the food web. For instance, species that are resources for the host might affect a typical parasite through the values of *b* and *d* in Box 2. Because such species may have their own dynamics, it can be difficult to predict indirect effects and feedbacks, restricting real world application of such simple models to infectious disease dynamics. It might be possible to ask how network structure affects things such as parasite persistence, host specialization and the potential for trophic transmission.

Some parasites might exploit many different hosts in the web, making it unclear which parameter values to use in disease models. The diversity of hosts in a food web can affect the *R*_0_ of pathogens (Dobson 2004) and typical parasites (P.A. Marquet, J.X. Velasco Hernandez, in prep.). Addressing this question requires analysis of an aspect of the food web, the ‘who acquires infection from whom’ (WAIFW) matrix (Anderson & May 1985). Expanding the elements of the WAIFW calculates a matrix, **G**, whose elements quantify the *R*_0_ for each interacting species pair (Diekmann *et al.* 1990). The spectral radius, or dominant eigenvalue, of **G** corresponds to the *R*_0_ of the parasite in a given food web. Such shortcuts to understanding diseases in communities of hosts could help integrate parasites into food webs.

Interactions between parasitism and predation can alter infection dynamics (Ostfeld & Holt 2004). For parasites that cannot survive the consumption of their host, predation reduces the availability of susceptible hosts (*H*), leading to a decrease in *R*_0_. Selective predation on heavily parasitized prey can exacerbate this effect by removing highly infectious hosts from the population (increasing α), provided the parasites die along with their hosts (Duffy *et al.* 2005). Furthermore, predation and parasitism on the free-living stages of parasites can increase the mortality (σ) of the parasite before it gets inside the host (Thieltges *et al.* 2008). Many of the most important effects of predation on parasitism will be indirectly mediated through food webs. For example, where spiny lobsters (predators) are common, they limit the density of sea urchins (prey), thereby reducing the frequency of bacterial epidemics in the urchin population (Lafferty 2004). Hence, the topology and dynamics of food webs may play an important role for the transmission success of parasites. Food webs can help us to explain the potential role of these links for parasite transmission and population dynamics.

By explicitly incorporating a broad species community, multiple trophic levels, and both parasite–host and predator–prey linkages, food-web models could prove valuable in understanding the direct and indirect responses of parasites to anthropogenic change. Usually, the loss of free-living biodiversity will result in a reduction in the diversity of parasites. For instance, trematodes are vastly reduced or absent from acidified systems because the required molluscan intermediate hosts cannot survive at low pH (Marcogliese & Cone 1996). Indirect effects are more difficult to predict outside a food-web framework. The loss of predators (coyotes, foxes, etc.) associated with human population expansions may indirectly increase Lyme disease cases in humans from the Northeast USA. Predators normally control the density of mice, which, as highly competent hosts, play a particularly important role in transmitting bacterium responsible for Lyme disease, *Borrelia burgdorferi* (Ostfeld & Holt 2004).

Other anthropogenic impacts may affect infectious disease from the bottom up. Eutrophication in aquatic systems is consistently associated with higher rates of parasitism (Lafferty 1997). In the simplest scenario, nutrients might directly supplement opportunistic pathogens, enhancing their transmission rate (β) and pathology (α) (Voss & Richardson 2006). Nutrient-enrichment may increase host density (*H*), particularly for herbivores, which benefit directly from heightened primary production. Increases in host density, in turn, can promote parasite transmission and *R*_0_. By increasing the encounter rate between susceptible hosts and infectious parasites, nutrient enrichment can also increase the production of parasite free-living stages (λ) and decrease the death rate of infected hosts (*d* and α), each of which will further promote *R*_0_ (e.g. Johnson & Carpenter 2008). Eutrophication also increases the density of herbivorous snails (the first intermediate host of trematodes) and the per-snail release of infectious cercariae, leading to an indirect increase in the trematode *Ribeiroia ondatrae*, which is a major cause of amphibian limb deformities (Johnson & Carpenter 2008).

## Conclusion

Initial studies indicate that there is considerable potential to learn about ecosystems by putting parasites into food webs. Obviously, determining general patterns for parasites in food webs will require repairing existing food webs currently missing parasites, developing food webs for systems where we currently have a good understanding of parasite diversity and considering parasites when constructing new food webs. We will also need to make room in food-web theory for the inclusion of parasites. This may be as simple as letting little things eat big things in models used to test for structure, or it may be as complicated as determining the range of consumer-resource models needed to expand dynamic food webs. The latter will benefit from a clearer understanding of allometric scaling relationships for parasites. In the process, numerous difficulties, such as how to accommodate multiple life-history stages, require resolution. A potential larger payoff to society for putting parasites into food webs will be a better understanding of infectious disease dynamics. Marcogliese & Cone's (1997) plea for parasites in food webs might then receive an answer.
